# SIVdrl detection in captive mandrills: are mandrills infected with a third strain of simian immunodeficiency virus?

**DOI:** 10.1186/1742-4690-1-36

**Published:** 2004-11-01

**Authors:** Antoinette C van der Kuyl, Remco van den Burg, Mark J Hoyer, Rob A Gruters, Albert DME Osterhaus, Ben Berkhout

**Affiliations:** 1 Department of Human Retrovirology, Academic Medical Centre, University of Amsterdam, Meibergdreef 15, 1105 AZ Amsterdam, The Netherlands; 2 Artis, Plantage Kerklaan 38–40, 1018 CZ Amsterdam, The Netherlands; 3 Department of Virology, Erasmus Medical Centre, P.O Box 1738, 3000 DR Rotterdam, The Netherlands

## Abstract

A pol-fragment of simian immunodeficiency virus (SIV) that is highly related to SIVdrl-pol from drill monkeys (*Mandrillus leucophaeus*) was detected in two mandrills (*Mandrillus sphinx*) from Amsterdam Zoo. These captivity-born mandrills had never been in contact with drill monkeys, and were unlikely to be hybrids. Their mitochondrial haplotype suggested that they descended from founder animals in Cameroon or northern Gabon, close to the habitat of the drill. SIVdrl has once before been found in a wild-caught mandrill from the same region, indicating that mandrills are naturally infected with a SIVdrl-like virus. This suggests that mandrills are the first primate species to be infected with three strains of SIV: SIVmnd1, SIVmnd2, and SIVdrl.

## Findings

**Figure 1:**
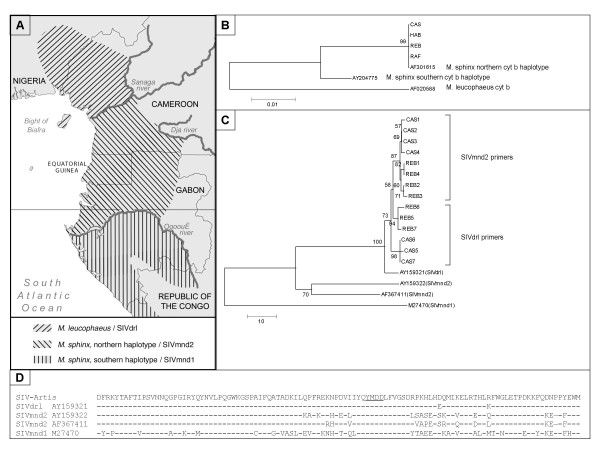
**A) **Geographic distribution of the genus *Mandrillus*, based upon mitochondrial haplotypes (adapted from [10]), and the SIV strains they harbour. **B) **Phylogenetic tree generated with Kimura-2-parameter distances and the NJ option of the MEGA package , from 267 nt of the mitochondrial cytochrome b gene of the four captive mandrills and reference sequences from GenBank (accession numbers are indicated). **C) **Phylogenetic tree generated with the NJ option of MEGA, based upon Kimura-2-parameter distances of SIVpol nucleotide fragments from captive mandrills CAS and REB, and reference sequences for SIVdrl, SIVmnd1, and SIVmnd2, respectively. Numbers shown are bootstrap confidence levels (BCL). **D) **SIV-pol amino acid consensus sequence from captive mandrills compared with homologous sequences from SIVdrl and SIVmnd2, respectively. The translated SIV-Artis sequence is the consensus sequence of 14 PCR clones derived from two animals. The YMDD motif within the catalytic core of the RT enzyme is underlined.

To date over 30 strains of simian immunodeficiency virus (SIV) have been isolated from African primate species and sequenced [1]. Mandrills (*Mandrillus sphinx*) are quite exceptional among African monkeys in that they harbour two distinct SIV strains, designated SIVmnd1 and SIVmnd2, with a separate geographic distribution [2,3] (see also Figure 1A). SIV infections are mostly non-pathogenic in their natural hosts. SIVmnd1, despite high virus levels in chronically infected mandrills, has only a small effect on the T-cell counts, and primary infection does not induce clinical symptoms [4,5]. However, two cases of immunodeficiency were reported in mandrills after long-term (>18 years) SIV infection [6]. In 2003, a 20-year old male captive mandrill (mandrill CAS) housed at Artis Zoo (Amsterdam, The Netherlands), suffering from heart failure and poor general condition, was found to be positive for serum SIV antibodies. Inspection of the other three mandrills of his group, a ten-year old female (mandrill REB) and their offspring (mandrills RAF, 3 years old and HAB, 2 months old), showed that the female and one of the offspring (HAB) were also SIV antibody-positive.

Both EDTA-plasma and PBMC (isolated with the OptiPrep system (Nycomed, Oslo, Norway)) was obtained from the animals for further analysis. Our goal was to investigate whether the monkeys were virus carriers, and which strain of SIV they harboured. To detect both SIVmnd1 and SIVmnd2, we designed two nested primers sets based on published pol gene sequences that amplify an RT fragment of 282 nucleotides (Table 1). Nucleic acids were isolated from PBMC by a procedure using silica and guanidium thiocyanate [7]. cDNA was synthesized with the 3'primer and AMV-RT (Roche Diagnostics, Penzberg, Germany). PCR amplifications were performed using the following protocol: denaturation for 5 min at 95°C and amplification for 35 cycles (first PCR) or 25 cycles (second PCR) of 1 min at 95°C, 1 min at 55°C, and 2 min at 72°C, followed by an extension of 10 min at 72°C. Products were cloned with the TOPO TA cloning kit (Invitrogen, San Diego, Calif.). Sequencing of at least four clones per sample was done with the Bigdye Terminator Cycle Sequencing kit and an ABI 377 automated sequencer (both from ABI, Foster City, Calif.), using M13 forward and M13 reverse primers. For species identification, fragments of the mitochondrial 12S and cytochrome B genes were amplified [8], and sequenced.

**Table 1:** PCR primers used to amplify *Mandrillus *SIV-pol

Primer	Sequence ('5→'3)	Description	Fragment size
SIVmnd1A	AGATATAGGGGATGCCTATT	5' first primer	A-B = 356 nt
SIVmnd1B	TCTTCCACTTATCTGGGTGT	3' first primer	
SIVmnd1C	AGATTATAGACCCTATACTGC	5'second primer	C-D* = 282 nt
SIVmnd1D	CATCCAATGAAAGGGAGGTTC	3' second primer	
SIVmnd2A	GGACATAGGGGATGCCTATT	5' first primer	A-B = 356 nt
SIVmnd2B	CTGTCCATTTCTTTGGGTGC	3' first primer	
SIVmnd2C	GGACTTTAGAAAGTACACTGC	5'second primer	C-D* = 282 nt
SIVmnd2D	CATCCACTCAAAGGGAGGTTC	3' second primer	
SIVdrlA	GGATGTAGGTGATGCCTATT	5' first primer	A-B = 356 nt
SIVdrlB	CTGTCCACTTCTTTGGATGC	3' first primer	
SIVdrlC	= SIVmnd2C	5'second primer	C-D* = 282 nt
SIVdrlD	CATCCATTCATAAGGAGGATTG	3' second primer	

* corresponding to nucleotides 2503–2784 of SIVdrl (acc. no. AY159321)

Mitochondrial 12S and cytochrome B sequences were identical in all four animals and confirmed that the monkeys were *M. sphinx *[9,10]. In addition, the cytochrome B sequences were indistinguishable from the recently described northern mandrill haplotype (Figure 1B) [10], suggesting that the captive animals descended from founders originating from a locale north of the Ogooué River (see Figure 1A). SIV-pol fragments could be amplified from PBMC of both adult mandrills CAS and REB with the primer set specific for SIVmnd2, but not with SIVmnd1 specific primers. However, analysis of the cloned fragments showed that these were 96–97% identical to SIVdrl-1FAO (GenBank acc. no. AY159321) isolated from a drill monkey (*Mandrillus leucophaeus*), with a lower sequence identity to SIVmnd2 (± 85% to GenBank acc. no. AF367411), and to SIVmnd1 (<64% to GenBank acc. no. M27470). Although SIVmnd2 and SIVdrl are more closely related to each other than the two SIVmnd strains, SIVdrl has several mismatches with the PCR primers designed for detection of SIVmnd2. This could explain why one seropositive animal was tested as PCR-negative. Therefore, we designed a new primer set that amplifies the same gene fragment based on the SIVdrl sequence (Table 1). Reanalysis of the mandrill PBMC samples with this drill-specific primer set again resulted in only two positive samples from the two adult animals: mandrills CAS and REB. Sequence analysis confirmed the high similarity to SIVdrl-1FAO (97%), and a lower similarity to SIVmnd2 (87%). SIV pol fragments from both mandrills were 98–99% similar to each other. Clones obtained from a single animal with the two primer sets were not identical to each other (98–99% identity), suggesting that each PCR amplified a subset of the virus population (Figure 1C).

Mandrills presently inhabit Cameroon, Gabon, and the southwestern part of the Republic of Congo. Two mitochondrial haplotypes are described in this species, separated by the Ogooué River in Gabon [10]. Interestingly, the distribution of mandrill SIV strains follows approximately the same geographic distribution, with SIVmnd1 being present in the southern part of the mandrill range, and SIVmnd2 in the northern part. Drill monkeys are found in Nigeria and Cameroon separated from the mandrill territory by the Sanaga River (Figure 1A). Mandrills and drills are currently believed to be non-sympatric, but it is not unlikely that the situation was different in the past.

SIVmnd2 and SIVdrl are closely related, and both are equidistant from SIVmnd1. SIVmnd2 is found in northern mandrills, which are closest to the current drill habitat. A wild-caught mandrill from south Cameroon was found to harbour a SIVdrl virus strain [3,11], suggestive of cross-species transmission [11]. Multiple cross-species transmissions are now believed to obscure the evolution and distribution of SIV strains in African primate species [1]. SIV cross-species transmissions are ongoing, and African green monkey strains have recently been detected in patas monkeys and baboons [1,12,13], species that are found in close proximity to each other.

All four mandrills examined here were born in captivity. Male CAS was born in 1983 at the now closed Wassenaar Zoo (The Netherlands), and moved to Artis Zoo in 1986. The female, REB, was born in Budapest Zoo (Hungary), and moved to Artis Zoo when she was 5 years old. Their offspring, RAF and HAB, were both born in Amsterdam. Exposure to drills during their lifetime is unlikely as none of the zoos kept drills (*M. leucophaeus*) at any time. Drills are rare in European zoos, and only the zoos of Nikolaev (Ukraine) and Saarbruecken (Germany) reported keeping both drills and mandrills in a 1992 survey [14]. So, it is improbable that the Artis mandrills acquired SIVdrl from a recent contact with captive drills. Another way of acquiring a drill SIV strain could be if one of the monkeys was actually a hybrid between a drill and a mandrill. Hybridisation between different species of Cercopithecinae is possible, and offspring is sometimes fertile depending upon the exact species. The genus *Mandrillus *cannot hybridise in the wild, as the habitats of the two species do not overlap, but it does so in captivity. The morphologic differences between female drills and mandrills are less obvious than those between males and are mainly noticeable in the colouration of the muzzle and the size of the animal. A single hybrid *M. sphinx *× *M. leucophaeus *has been reported from Vienna Zoo, Austria, in a 1992 survey [14], and two hybrid *M. leucophaeus *× *M. sphinx *were described from a Wildlife Rescue Centre in Cameroon [15]. Mitochondrial sequencing as performed in this study cannot alone be used to resolve hybridisation, as it only characterises the mother lineage. The male mandrill is, however, unlikely to be a hybrid as its description fits exactly that of a male mandrill. The female was also listed in the Artis Zoo database records as a non-hybrid. She was registered with the European studbook programme (ESB) for mandrills supervised by the Budapest Zoo, Hungary, and was born from registered mandrill parents.

Because SIVdrl was also found in a wild-caught Cameroonian mandrill [3,11], it is plausible that a SIVdrl-like virus is naturally present in mandrills, making them the only primate species that is naturally infected with three strains of SIV. The presence of SIVdrl in one of the two adult captive mandrills occurred probably through transmission from a wild ancestor, and this animal probably infected the other mandrill once joined in Artis Zoo. Sexual and mother-to-child transmission of SIV in mandrills have been reported to be rare [16,17]. Here, in offspring born to a SIV-positive mother SIV could not be detected. The persistence of maternal antibodies could explain why the 2-month old young tested seropositive, although there is a possibility of the animal having a viral copy number below the detection limit of the PCR assay. Transmission of SIV between mandrills and drills could have taken place either by biting or sexual contacts. SIVmnd1 is mainly transmitted between males during aggressive contacts [17], and might also be transmitted when fighting off other receptive primates species. Sexual transmission could have occurred during interbreeding. Each of these possibilities requires an overlap of habitats, which could have existed in the past.

If we assume that SIVdrl(Artis) left Africa at least ten years ago (when the female was born in captivity), its genome conservation is remarkable: only 2 conserved amino acid differences separate the consensus pol-sequence from the SIVdrl reference sequence (Figure 1D). However, to gain a deeper insight into the characteristics and evolution of this virus strain, a full-length sequence, or at least additional sequences of the gag or env regions, would be required. Only then could it be determined whether the virus carried by the captive mandrills is really SIVdrl or a novel recombinant virus. Several recombinant SIVs have been described in naturally infected primate species (see: [1]). Unfortunately, further sequence analysis is difficult due to shortage of material as the monkeys were euthanized soon after the SIV antibody test results became known.

## Competing interests

The author(s) declare that they have no competing interests.

## Authors'contributions

ACvdK designed the study, analysed the sequences, and drafted the manuscript. RvdB carried out the PCR assays and performed the cloning and sequencing. MJH did the medical examinations and collected the blood samples. RAG carried out the SIV antibody assays. ADMEO and BB conceived of the study, and participated in its coordination.

## References

[B1] Bibollet-Ruche F, Bailes E, Gao F, Pourrut X, Barlow KL, Clewley JP, Mwenda JM, Langat DK, Chege GK, McClure HM, Mpoudi-Ngole E, Delaporte E, Peeters M, Shaw GM, Sharp PM, Hahn BH (2004). New simian immunodeficiency virus infecting De Brazza's monkeys (Cercopithecus neglectus): evidence for a cercopithecus monkey virus clade. J Virol.

[B2] Souquiere S, Bibollet-Ruche F, Robertson DL, Makuwa M, Apetrei C, Onanga R, Kornfeld C, Plantier JC, Gao F, Abernethy K, White LJ, Karesh W, Telfer P, Wickings EJ, Mauclere P, Marx PA, Barre-Sinoussi F, Hahn BH, Muller-Trutwin MC, Simon F (2001). Wild Mandrillus sphinx are carriers of two types of lentivirus. J Virol.

[B3] Takehisa J, Harada Y, Ndembi N, Mboudjeka I, Taniguchi Y, Ngansop C, Kuate S, Zekeng L, Ibuki K, Shimada T, Bikandou B, Yamaguchi-Kabata Y, Miura T, Ikeda M, Ichimura H, Kaptue L, Hayami M (2001). Natural infection of wild-born mandrills (Mandrillus sphinx) with two different types of simian immunodeficiency virus. AIDS Res Hum Retroviruses.

[B4] Onanga R, Kornfeld C, Pandrea I, Estaquier J, Souquiere S, Rouquet P, Mavoungou VP, Bourry O, M'Boup S, Barre-Sinoussi F, Simon F, Apetrei C, Roques P, Muller-Trutwin MC (2002). High levels of viral replication contrast with only transient changes in CD4(+) and CD8(+) cell numbers during the early phase of experimental infection with simian immunodeficiency virus SIVmnd-1 in Mandrillus sphinx. J Virol.

[B5] Pandrea I, Onanga R, Kornfeld C, Rouquet P, Bourry O, Clifford S, Telfer PT, Abernethy K, White LT, Ngari P, Muller-Trutwin M, Roques P, Marx PA, Simon F, Apetrei C (2003). High levels of SIVmnd-1 replication in chronically infected Mandrillus sphinx. Virology.

[B6] Pandrea I, Onanga R, Rouquet P, Bourry O, Ngari P, Wickings EJ, Roques P, Apetrei C (2001). Chronic SIV infection ultimately causes immunodeficiency in African non-human primates. AIDS.

[B7] Boom R, Sol CJ, Salimans MM, Jansen CL, Wertheim-van Dillen PM, van der Noordaa J (1990). Rapid and simple method for purification of nucleic acids. J Clin Microbiol.

[B8] Kocher TD, Thomas WK, Meyer A, Edwards SV, Paabo S, Villablanca FX, Wilson AC (1989). Dynamics of mitochondrial DNA evolution in animals: amplification and sequencing with conserved primers. Proc Natl Acad Sci U S A.

[B9] van der Kuyl AC, Kuiken CL, Dekker JT, Goudsmit J (1995). Phylogeny of African monkeys based upon mitochondrial 12S rRNA sequences. J Mol Evol.

[B10] Telfer PT, Souquiere S, Clifford SL, Abernethy KA, Bruford MW, Disotell TR, Sterner KN, Roques P, Marx PA, Wickings EJ (2003). Molecular evidence for deep phylogenetic divergence in Mandrillus sphinx. Mol Ecol.

[B11] Hu J, Switzer WM, Foley BT, Robertson DL, Goeken RM, Korber BT, Hirsch VM, Beer BE (2003). Characterization and comparison of recombinant simian immunodeficiency virus from drill (Mandrillus leucophaeus) and mandrill (Mandrillus sphinx) isolates. J Virol.

[B12] Jin MJ, Rogers J, Phillips-Conroy JE, Allan JS, Desrosiers RC, Shaw GM, Sharp PM, Hahn BH (1994). Infection of a yellow baboon with simian immunodeficiency virus from African green monkeys: evidence for cross-species transmission in the wild. J Virol.

[B13] van Rensburg EJ, Engelbrecht S, Mwenda J, Laten JD, Robson BA, Stander T, Chege GK (1998). Simian immunodeficiency viruses (SIVs) from eastern and southern Africa: detection of a SIVagm variant from a chacma baboon. J Gen Virol.

[B14] Wilde J, Klensang H, Schwibbe MH (1994). A census for captive primates in Europe and North Africa for 1992. PRIMATE REPORT.

[B15] Lacoste V, Mauclere P, Dubreuil G, Lewis J, Georges-Courbot MC, Rigoulet J, Petit T, Gessain A (2000). Simian homologues of human gamma-2 and betaherpesviruses in mandrill and drill monkeys. J Virol.

[B16] Georges-Courbot MC, Moisson P, Leroy E, Pingard AM, Nerrienet E, Dubreuil G, Wickings EJ, Debels F, Bedjabaga I, Poaty-Mavoungou V, Hahn NT, Georges AJ (1996). Occurrence and frequency of transmission of naturally occurring simian retroviral infections (SIV, STLV, and SRV) at the CIRMF Primate Center, Gabon. J Med Primatol.

[B17] Nerrienet E, Amouretti X, Muller-Trutwin MC, Poaty-Mavoungou V, Bedjebaga I, Nguyen HT, Dubreuil G, Corbet S, Wickings EJ, Barre-Sinoussi F, Georges AJ, Georges-Courbot MC (1998). Phylogenetic analysis of SIV and STLV type I in mandrills (Mandrillus sphinx): indications that intracolony transmissions are predominantly the result of male-to-male aggressive contacts. AIDS Res Hum Retroviruses.

